# Development of a Specifically Enhanced Enzyme-Linked Immunosorbent Assay for the Detection of Melamine in Milk

**DOI:** 10.3390/molecules16075591

**Published:** 2011-06-30

**Authors:** Hongtao Lei, Rui Su, Simon A. Haughey, Qiang Wang, Zhenlin Xu, Jinyi Yang, Yudong Shen, Hong Wang, Yueming Jiang, Yuanming Sun

**Affiliations:** 1 Guangdong Provincial Key Laboratory of Food Quality and Safety, South China Agricultural University, Guangzhou 510642, Guangdong Province, China; Email: hongtao@scau.edu.cn (H.L); suruisue@hotmail.com (R.S.); wang008.qiang@163.com (Q.W.); jallent@163.com (Z.X.); yjy361@163.com (J.Y.); shenyudong@scau.edu.cn (Y.S.); gzwhongd@163.com (H.W.); 2 Institute of Agri-Food and Land Use, Queen’s University Belfast, Belfast BT9 5AG, Northern Ireland, UK; Email: s.a.haughey@qub.ac.uk; 3 Key Laboratory of Plant Resources Conservation and Sustainable Utilization, South China Botanical Garden, Chinese Academy of Sciences, Guangzhou 510650, Guangdong Province, China

**Keywords:** melamine, ELISA, specificity, milk

## Abstract

An indirect competitive enzyme-linked immunosorbent assay (icELISA) with enhanced specificity for melamine in milk was developed. Three haptens of melamine with different spacer-arms were used to prepare different plate coating antigens. It was found that the icELISA show best sensitivity and specificity to melamine when using the coating antigen prepared by coupling 3-(4,6-diamino-1,6-dihydro-1,3,5-triazin-2-ylthio)propanoic acid (Hapten C) with ovalbumin (OVA). The 50% inhibitory concentration (IC_50_) value was 35.4 ng·mL^−1^, the limit of detection (LOD) was 8.9 ng·mL^−1^ and the detectable working range (20–80% inhibitory concentration) was from 14.9 to 108.5 ng·mL^−1^, respectively. Compared to the ELISA results previously reported, the developed icELISA in the present study showed a much lower cross-reactivity to cyromazine, a fly-killing insecticide widely used in vegetables and stables. Recoveries obtained from milk samples in this study were in agreement with those obtained using the HPLC-MS method, indicating the detection performance of the icELISA could meet the requirement of the residue limit set by the Codex Alimentarius Commission. Therefore, the developed immunoassay can be applied for the analysis of melamine presented in milk.

## 1. Introduction

**Figure 1:**
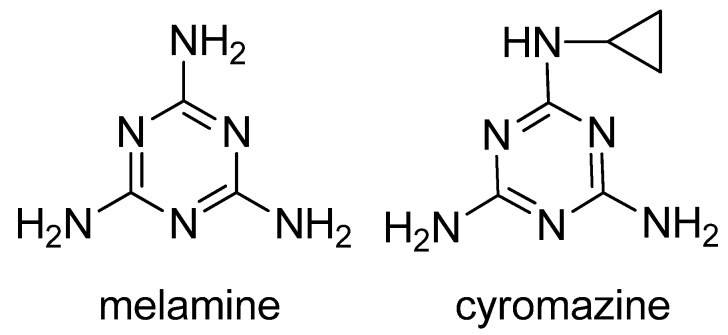
Structures of melamine and cyromazine.

Melamine (2,4,6-triamino-1,3,5-triazine, Figure 1) is a basic organic compound, with a chemical formula of C_3_H_6_N_6_, which has been widely used in polymer resins or as a raw material in the chemical industry. Melamine in combination with triazine or cyanuric acid forms insoluble melamine cyanurate crystals in the kidney, causing renal failure in animals and humans [1,2]. A safety limit of melamine ingestion has been officially set as 1 mg·kg^−1^ in powdered infant formula and 2.5 mg·kg^−1^ in other foods or animal feed by the Codex Alimentarius Commission (CAC) [3]. Due to the fact it is a nitrogen-rich compound, melamine could be illegally added into food materials to fraudulently increase the protein content. In 2008, continuous consumption of the melamine-contaminated milk and infant milk powder caused renal stones in thousands of children in China [2]. Therefore, it is important to be able to rapidly monitor potential melamine residues in food-related products.

Several methods have been developed recently to detect melamine based on gas or liquid chromatography [4,5], capillary electrophoresis [4,6], infrared [7] or nuclear magnetic resonance spectroscopy [8], molecular imprint [9], colorimetric detection [10] and biosensors [11,12]. Although each is accurate and reliable, some of these methods are expensive, laborious and time-consuming. Antibody-based immunoassay remains a good method for accurate, sensitive, routine, and portable detection [13]. Thus, development of a practical immunochemical test to assess melamine contamination prior to consumption of dairy food is needed. 

Several analytical methods involving melamine immunoassays have been reported. However, in these reports, the cross-reactivity (CR) to the insecticide cyromazine was too high or the CR was not evaluated well [14,15,16,17]. Cyromazine (N2-cyclopropyl-1,3,5-triazine-2,4,6-triamine, Figure 1) is a triazine pesticide used for fly control in crop production and animal feed to inhibit insect growth, whereas melamine is the major metabolite of cyromazine. Thus, cyromazine and melamine can exist simultaneously in animal-derived food [18]. Due to the structural similarity to melamine, cyromazine is a major potential matrix factor which is not expected in the immunochemical analysis of melamine. In the previous ELISA study, we used 3-(4,6-diamino-1,6-dihydro-1,3,5-triazin-2-ylamino) hexanoic acid as immunizing hapten to obtain an antibody against melamine. Unexpectedly, the antibody exhibited a strong CR (267.6%) to cyromazine [16]. A recently reported ELISA also demonstrated a high CR (59%) to cyromazine [19]. Thus, immunoassay with high CR is suitable for multi-residue analysis, but it is not expected in a single-analyte specific analysis [20]. To our best knowledge, no ELISAs focus on lowering the CR for cyromazine has been reported.

In this study, three haptens with different spacer-arms were coupled to ovalbumin (OVA) for coating antigens and the effects of homologous and heterologous coating antigens on sensitivity and specificity of ELISA were studied. It was interesting to find that when using the coating antigen prepared by coupling 3-(4,6-diamino-1,6-dihydro-1,3,5-triazin-2-ylthio) propanoic acid (Hapten C), the sensitivity for melamine was improved while the CR for cyromazine was significantly decreased. The specific icELISA was further applied to detect melamine in spiked milk samples, and the results were confirmed by HPLC-MS method.

## 2. Results and Discussion

### 2.1. Conjugate Preparation

Some commercial ELISA kits has been available for the immunoassay of melamine [17,18,20,23,24,25] and there have been four published papers describing the hapten synthesis: the *tert*-butyl bromoacetate [17], glutaradehyde [21], succinic ahydride [19] and 2-chloro-4,6-diamino-1,3,5-triazine (CAAT) methods [16]. The CATT method is easy to carry out, but the resultant antibody has been already verified to strongly recognize both melamine and cyromazine [16], which is not unsuitable for a specific immunoassay to a single analyte. The antibody resulting from the succinic anhydride method also demonstrated high recognization ability to cyramazine [19], and other papers have given no data on antibody binding to cyromazine.

**Figure 2:**
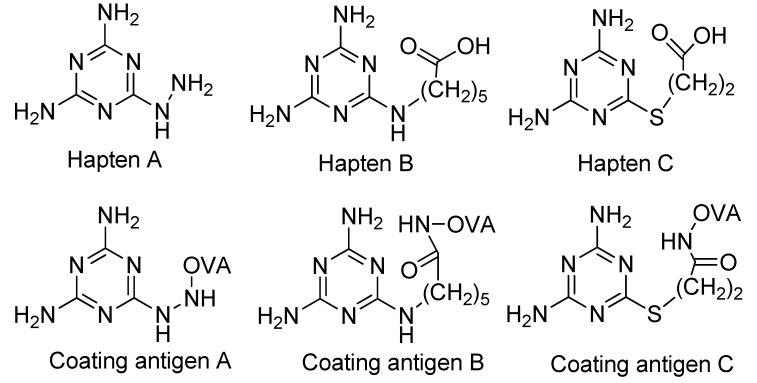
Chemical structures of haptens and coating antigens.

**Figure 3:**
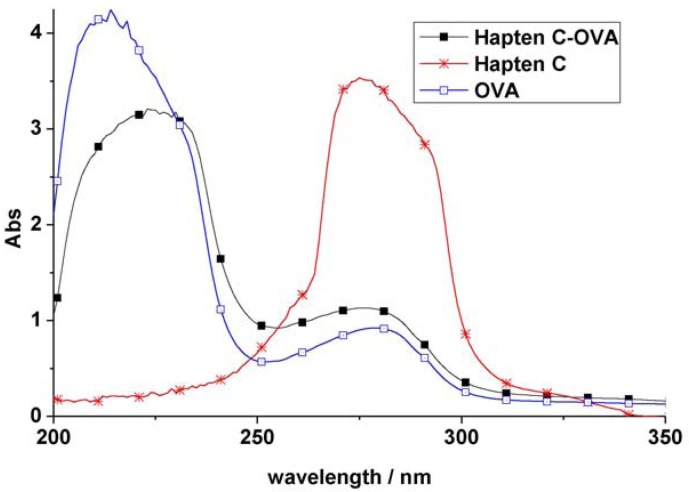
Verification of conjugation for typical hapten-protein conjugate.

However, in the development of ELISA for small molecules, the use of heterologous coating antigens was reported to not only improve the sensitivity but also to change the specificity [20]. To obtain coating antigens, In the present study Haptens A, B and C (Figure 2) were covalently attached to OVA using the water soluble carbodiimide method with one condensation step. The UV absorbance properties of the protein conjugates allowed the verification of the coupling reaction [22]. The absorbance wavelength of the peaks for Haptens A, B and C appeared at 264, 241 and 275 nm, respectively, and the spectral characteristics of the conjugates were similar. As an example, the UV absorption spectra of the coating antigen C (Hapten C-OVA), OVA and Hapten C are shown in Figure 3. Quantitative changes in the 260–280 nm spectral region indicated a successful covalent attachment [22], and, thus, the conjugation between hapten C and OVA can be concluded to be successful. Clearly, the use of one condensation step to obtain conjugates as described herein is simpler than other reported methods requiring two reaction steps from hapten to conjugate [16,17].

### 2.2. Coating Antigen Selection

**Figure 4:**
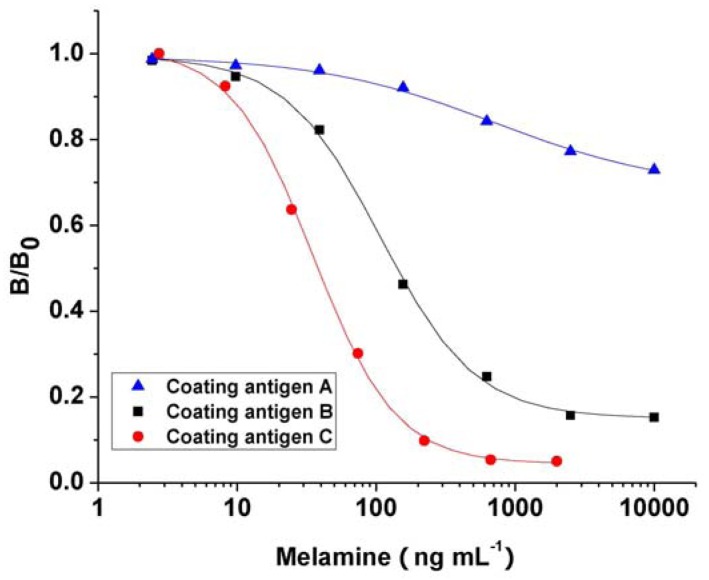
Typical calibration curves for melamine based on different coating antigen.

The antibody binding ability to three plate coating antigens were evaluated by icELISA. Figure 4 shows the typically optimized calibration curves for melamine based on different coating antigens while the analytical characteristics of plate coating Antigen A as a homologous antigen and plate coating Antigens B and C as heterologous antigens are summarized in Table 1. Antibodies showed strong binding abilities to the three plate coating antigens, with titers ranging from 1/6,000 to 1/60,000. It is known that the ELISA format can influence the assay sensitivity while the heterogeneous coating antigen can often result in an antibody with a higher affinity towards the analyte than the coating antigen or tracer hapten [23]. In the present study, both homologous and heterologous coating antigens can be compared to select the best sensitivity between antibody and coating antigen. As expected, although the heterologous coating Antigens B and C showed lower titers than the homologous coating Antigen A, coating Antigens B and C exhibited much higher sensitivity (lower IC_50_ value and LOD), and their sensitivity of the developed icELISA can meet the requirement of CAC [3], which implied that the heterologous plate coating antigens significantly enhanced the assay sensitivity.

Somewhat surprisingly, fluorescein labeled Hapten B demonstrated a much higher sensitivity in a fluorescence polarization immunoassay (FPIA) study than fluorescein labeled Hapten C [24], however, the most optimal coating hapten in the present ELISA is plate coating Antigen C. Similar behavior was also observed in the detection of mycotoxin deoxynivalenol, in which the hapten in ELISA did not work well in a fluorescence polarization immunoassay, and, thus, a linker difference between tracer and fluorescein was possibly postulated [25]. Unfortunately, some attempts to develop FPIAs using antibodies from ELISA were not successful [26]. It suggested that various immunoassay formats may depend on different antibody kinetic characteristics for the optimal performance. As a result, the difference between ELISA and FPIA in this study clearly indicated that the design and choice of tracer or coating antigen exhibited a particular significance to detect melamine.

**Table 1:** Analytical characteristics for melamine based on different coating antigens.

Plate Coating Antigen	Coating Concentration (ng·mL^−1^)	Titer	A_max_	IC_50_ (ng·mL^−1^)	LOD (ng·mL^−1^)	*Working Range *(ng·mL^−1^)
A	22.2	6 × 10^4^	1.601	632.8	221.0	ND ^a^
B	16.7	6 × 10^3^	1.87	107.5	21.0	41.5–952.8
C	133.3	8 × 10^3^	1.42	35.4	8.9	14.9–108.5

^a^ ND represented that the 20-80% inhibitory concentration could not be obtained with the four-parameter logistic equation due to the irregular curve shape based on coating antigen A.

### 2.3. Cross-Reactivity

The cross-reactivity (CR) is not dependent on weight, but it is site and structure specific, with a molar dependent quantity [27]. For this purpose, the molar CRs of several related compounds were tested using melamine as the reference compound (CR = 100%). As shown in Table 2, when using plate coating antigen C as the competitive antigen, CRs for Hapten B and C, cyromazine, Hapten A and CAAT were 12.1, 18.0, 17.1, 10.7 and 6.2%, respectively. Further, CRs for ammeline, ammelide, cyanuric acid, cyanuric chloride and atrazine showed less than 0.01%. When plate coating antigen B was used as the competitive antigen, CRs for Hapten B and C, cyromazine, Hapten A and CAAT were 25.0, 13.2, 21.1, 9.8 and 5.2%, respectively. And CRs for ammeline, ammelide, cyanuric acid, cyanuric chloride and atrazine showed less than 0.01%. However, when the coating antigen A was used, the does-response curve could not to fit the four parameter logistic equation due to the exessively high IC_50_ value obtained. Therefore, the CR data based on the homogeneous coating antigen A could not be analyzed. 

**Table 2:** Cross-reactivity of antibody to related compounds based on different coating antigens.

Compound	Structure	Space-filling model	Coating antigen C		Coating antigen B	
			IC_50_ (nmol·mL^−1^)	CR%	IC_50_ (nmol·mL^−1^)	CR%
Melamine	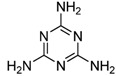	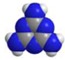	0.28	100	0.85	100.0
Hapten B	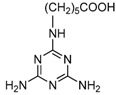	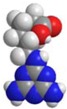	2.31	12.1	3.41	25.0
Hapten C	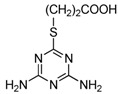	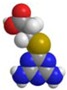	1.54	18.0	6.44	13.2
Cyromazine	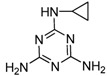	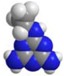	1.61	17.1	4.03	21.1
Hapten A	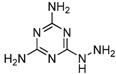	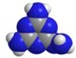	2.57	10.7	8.72	9.8
CAAT	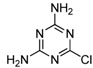	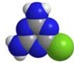	4.43	6.2	16.33	5.2
Ammeline	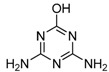	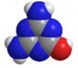	ND ^a^	≤0.01	ND	≤0.01
Ammelide	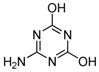	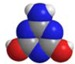	ND	≤0.01	ND	≤0.01
Cyanuric acid	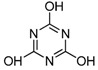	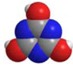	ND	≤0.01	ND	≤0.01
Cyanuric chloride	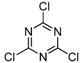	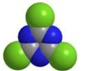	ND	≤0.01	ND	≤0.01
Atrazine	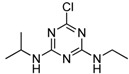	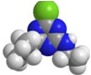	ND	≤0.01	ND	≤0.01

^a^ ND presented infinite IC_50_ values and could not be fitted with the four-parameter logistic equation.

However, it is clear that CR to cyromazine was decreased greatly using either coating antigen C or B. Using the coating antigen C, the CR to cyromazine in this present study decreased more than 10-fold compared with that of the ELISA reported by Lei *et al.* [16], and 3.5-fold compared with that of the ELISA reported by Liu *et al.* [19], while the specificity to cyromazine was better than that using coating Antigen B. The results confirmed that the heterologous combination between immunogen and plate coating antigen could greatly improve the specificity. Moreover, even using the same coating antigen C, the CR% to cyromazine, Haptens B and C were much lower than that of previous report [16], which should be attributed to the immunizing hapten difference that resulted in the different antibody. Similarly, the difference of antibody performance should also be responsible for the LOD difference of the methods in this study and the previous.

To better explain the CRs of the obtained antibody, the space filling models of melamine analogues were obtained, as shown in Table 2. Usually, the antibody can recognize the immunizing hapten with high CR. However, the CR to the immunizing hapten (Hapten A) in the present study was only about 10% (Table 2). Since Hapten A was coupled to carrier protein through the –NH_2_ group, the –NH_2_ group might not participate in stimulating antibody formation. As a result, the obtained antibody showed poor recognition ability to free Hapten A due to the presence of –NH_2_ group. The lower CRs for Hapten B, C and cyromazine could be postulated to the steric hindrance of the side chain, because steric effect is a well known factor to influence the antibody-hapten binding [28]. When the –NH_2_ group of melamine was substituted by a chlorine atom (CATT and cyanuric chloride) or a hydroxyl (ammeline, ammelide and cyanuric acid), the CRs decreased significantly (Table 2). The reasons should be due to the change of their electronic properties, which is also an important factor to influence the antibody-hapten binding [28]. 

Using coating antigen C, the 17.1% CR of cyromazine was a bit high, but it could be acceptable in a screening method due to the merit of its rapidity and simplicity [29,30]. Although Hapten C and CATT showed 18.0% and 6.2% cross-reactivity, they both are usually artificial and, therefore, have seldom chance to emerge in milk to cause the matrix effects. As a result, the developed ELISA can meet the specificity requirement for the screening assay of melamine.

### 2.4. Analysis of Spiked Samples

The analytical characteristics of an immunochemical technique can be significantly influenced by the various components existing in some complicated matrices such as fat [9,10]. The matrix effects were estimated in this study using a brand of liquid milk purchased from a local market, which was confirmed to be negative by the HPLC-MS method. For the icELISA, the raw milk sample was centrifuged to remove milk fat to obtain the clear middle liquid layer. The absorbance of the 6-fold diluted middle liquid layer was found to be almost same to that of the PBST (data not show), indicating no obvious matrix effect in the middle liquid layer after defatting and dilution. Accordingly, the 6-fold dilution was selected for the subsequent experiments.

**Table 3:** Recovery of melamine from the spiked samples by icELISA.

Sample	Added Levels (ng·mL^−1^)	Observed Value (ng·mL^−1^)	Recovery (%, *n *= 3)	Recovery Mean (%)	CV (%)	CV Mean (%)
Milk	120.0	116.3 ± 7.4	96.9 ± 6.2	93.4	6.4	6.0
	240.0	214.5 ± 5.3	89.4 ± 2.2		2.5	
	360.0	340.4 ± 13.8	94.6 ± 3.8		4.0	
	480.0	435.0 ± 18.2	90.6 ± 3.8		4.2	
	540.0	516.5 ± 66.8	95.7 ± 12.4		12.9	

Milk samples were spiked with melamine at different concentration levels to determine the recoveries (Table 3). It was found that the icELISA demonstrated a satisfactory recovery in the range of 89.4–96.9%, which was in good agreement with the amounts spiked, with the mean coefficient of variation of 6.0%.

### 2.5. Validation by HPLC-MS

**Figure 5:**
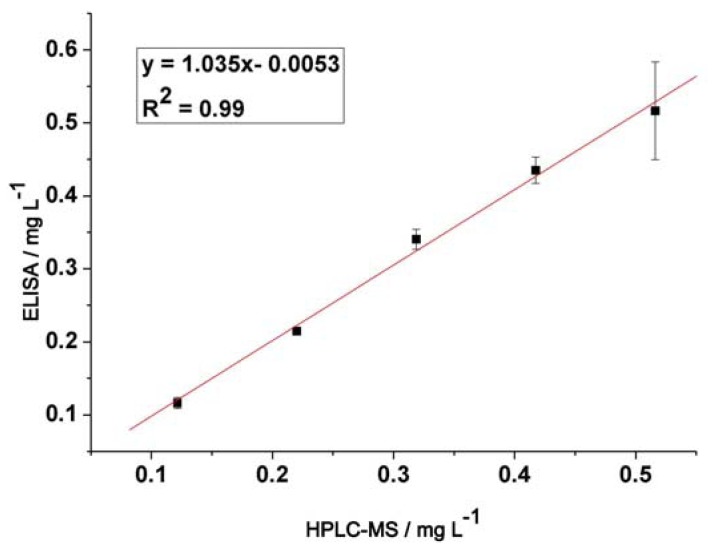
Correlation of icELISA with HPLC-MS analysis for milk samples (n = 3).

To confirm the accuracy of the icELISA in the present study, five milk samples (from the same brand of milk) spiked with different levels of melamine (120, 240, 360, 480 and 540 ng·mL^−^^1^) were determined by both the ELISA and the HPLC-MS method. As shown in Figure 5, an excellent linear correlation between icELISA and HPLC-MS (*r^2^* = 0.99 and slope 1.035) was obtained, which indicated the reliability of the proposed icELISA for the detection of melamine in milk sample.

## 3. Experimental

### 3.1. Reagents

General reagents and organic solvents were of analytical grade, unless specified. Melamine, bovine serum albumin (BSA), ovalbumin (OVA), 1-ethyl-3-(3-dimethylaminopropyl)carbodiimide hydrochloride (EDC), complete and incomplete Freund’s adjuvants were purchased from Sigma Co. (St. Louis, MO, USA) while HRP-conjugated goat anti-rabbit IgG was obtained from Boster Biotech Co. Ltd. (Wuhan, China). Polystyrene ELISA plates were provided from Jincanhua Co., Ltd. (Shenzhen, China) and 6-hydrazinyl-1,3,5-triazine-2,4-diamine (Hapten A) was obtained from Shanghai Chaoyan Biotechnology Co. Ltd. (Shanghai, China) while 3-(4,6-diamino-1,6-dihydro-1,3,5-triazin-2-ylthio)propanoic acid (Hapten C), 6-(4,6-diamino-1,6-dihydro-1,3,5-triazin-2-ylamino) hexanoic acid (Hapten B) and the antibody used in this study were prepared from Hapten A using a previously decribed method [24]. 

### 3.2. Instrumentation

A UV-3010 spectrophotometer (Hitachi, Japan) was used and ELISA plates were washed with a Multiskan MK-2 microplate washer (Thermo Labsystems, USA). Absorbance was measured at a wavelength of 450 nm using the Wallac 1420 VICTOR3 multiable counter (Perkin Elmer Ltd., US).

### 3.3. Preparation of Hapten-Protein Conjugates

Plate coating antigen A (Hapten A–OVA) (Figure 2) was obtained using the EDC method [24]. Briefly, Hapten A (3 mg, 20 µmol) dissolved in absolute DMF (100 µL) was added dropwise with stirring to BSA (10 mg) in 0.9% (m/v) NaCl (1.9 mL), followed by the addition of EDC (5 mg, 25 µmol), and then kept for 2 h at room temperature. Finally, the conjugates were dialyzed against 0.9% (m/v) NaCl solution for two days with three changes per day. The dialyzed product was collected as plate coating Antigen A and then stored at −20 °C until use. The scan diagrams of the hapten, OVA and the conjugate were obtained on a UV-Vis spectrophotometer to identify the conjugation [22]. Plate coating Antigens B and C (Figure 2) were prepared by the active ester method previously described [16].

### 3.4. ELISA Protocol

The melamine concentration was determined by the indirect competitive ELISA (icELISA). Briefly, 96-well polystyrene microtiter plates were coated (100 µL per well) with hapten-OVA in carbonate buffer (1.59 g·L^−1^ Na_2_CO_3 _and 2.93 g·L^−1^ NaHCO_3_, pH 9.6), and then incubated at 37 °C overnight. After washing with 2 times using phosphate-buffered saline with Tween 20 (PBST, 8 g·L^−1^ NaCl, 0.2 g·L^−1^ KH_2_PO_4_, 1.2 g·L^−1^ Na_2_HPO_4_ and 0.2 g·L^−1^ KCl containing 0.05% Tween 20, pH 7.4), these plates were added to 200 µL of blocking buffer per well, then incubated for 2 h at 37 °C and finally washed twice with PBST. Melamine standards were dissolved in PBST, applied to the plate at 50 µL per well, and finally 50 µL of the diluted antiserum in PBST per well was added. After mixing for 30 s, the plate was incubated for 40 min at 37 °C and then washed five times with PBST. Goat anti-rabbit-HRP was diluted 1:5,000 and then added with 100 µL per well. After 30 min of incubation at 37 °C the plate was washed five times with PBST solution. Finally, TMB solution was added to the wells, with 100 µL per well and then incubated for 10 min. The reaction was stopped by addition of 50 µL of 2 M H_2_SO_4_ per well, and then the absorbance was recorded at 450 nm. Competitive curves were obtained by plotting the normalized signal B/B_0_ against the logarithm of analyte concentration with a four parameter logistic equation [16], with B_0 _being the signal without analyte and B being the signal of each concentration of analyte. The 50% inhibitory concentration *(IC_50_)* and the limit of detection (LOD) defined as 10% inhibitory concentration (IC_10_), and detectable concentration range (IC_20_–IC_80_) were obtained from these calibration curves.

### 3.5. Specificity

Specificity of the optimized assay was tested by measuring the cross-reactivity using competitors, and then the IC_50_ values were obtained to calculate the cross-reactivity using the following formula. IC_50_ was the concentration of analyte inhibiting 50% of the assay signal:

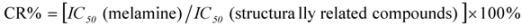
(1)

### 3.6. Analysis of Spiked Samples

To evaluate the recovery of the icELISA developed, melamine-free raw milk (1 mL) was spiked at 120, 240, 360, 480 and 540 ng of melamine standard in PBST, respectively. The melamine-spiked milk was centrifuged for 15 min at 10,000 g and 4 °C to remove the milk fat. The clear liquid layer in the middle was diluted appropriatly in PBST to eliminate the matrix effect prior to an icELISA. To validate the accuracy of ELISA, each sample was also analyzed by HPLC-MS. The HPLC-MS analysis procedure was the same as described by Yin [17]. The HPLC-MS analysis was carried out by Guangdong Testing Institute for Product Quality, Shunde, Guangdong Province, China. 

## 4. Conclusions

In this study, three plate coating antigens were prepared and used for developing an ELISA to achieve a specificity-enhanced assay for the detection of melamine. Compared with the previously described indirect ELISA, the icELISA in this study using the heterologous combination of coating antigen and antibody was greatly improved the specificity and sensitivity. Melamine-spiked milk samples were determinable by the icELISA with excellent recovery and CV. Also, the developed method showed a good agreement with the HPLC-MS method. Therefore, this developed ELISA can be applied for the rapid detection of melamine in milk.
